# Genome-Wide Prediction of the Polymorphic *Ser* Gene Family in *Tetrahymena thermophila* Based on Motif Analysis

**DOI:** 10.1371/journal.pone.0105201

**Published:** 2014-08-18

**Authors:** Patrath Ponsuwanna, Krittikorn Kümpornsin, Thanat Chookajorn

**Affiliations:** 1 Department of Biochemistry, Faculty of Science, Mahidol University, Bangkok, Thailand; 2 Center of Excellence in Malaria, Faculty of Tropical Medicine, Mahidol University, Bangkok, Thailand; Washington State University, United States of America

## Abstract

Even though antigenic variation is employed among parasitic protozoa for host immune evasion, *Tetrahymena thermophila*, a free-living ciliate, can also change its surface protein antigens. These cysteine-rich glycosylphosphatidylinositol (GPI)-linked surface proteins are encoded by a family of polymorphic *Ser* genes. Despite the availability of *T. thermophila* genome, a comprehensive analysis of the *Ser* family is limited by its high degree of polymorphism. In order to overcome this problem, a new approach was adopted by searching for *Ser* candidates with common motif sequences, namely length-specific repetitive cysteine pattern and GPI anchor site. The candidate genes were phylogenetically compared with the previously identified *Ser* genes and classified into subtypes. *Ser* candidates were often found to be located as tandem arrays of the same subtypes on several chromosomal scaffolds. Certain *Ser* candidates located in the same chromosomal arrays were transcriptionally expressed at specific *T. thermophila* developmental stages. These *Ser* candidates selected by the motif analysis approach can form the foundation for a systematic identification of the entire *Ser* gene family, which will contribute to the understanding of their function and the basis of *T. thermophila* antigenic variation.

## Introduction

*Tetrahymena thermophila* is a single-celled ciliate found in temperate freshwater [1], [2]. *T. thermophila* naturally feeds on bacteria, but it can also grow in media under laboratory conditions [3]. It has two nuclei, a micronucleus (MIC) as a germ line and a macronucleus (MAC) as a source specific for gene expression [4]. When food is abundant, *T. thermophila* reproduces asexually, but starvation induces conjugation between different mating types [4]. Nuclei developed from parental MICs are exchanged between two mating pair to produce new MIC and MAC. Nucleus destined to become a new MAC undergoes DNA rearrangements, including deletion of internal eliminated sequences (IES), removal of repetitive sequences and chromosome breakage at specific sites [4]. During the development of the new MAC, the parental MAC becomes degraded [4], [5]. This unique biology of *T. thermophila* makes it an important model organism leading to seminal discoveries in the field of molecular biology [2].

*T. thermophila* cell membrane is covered by a surface protein known as immobilization antigen (i-ag) [6], [7], as incubation with antibodies against i-ag causing *T. thermophila* to cease its movement, hence the name. Various subtypes of *T. thermophila* i-ag have been described based on immobilization assays with specific antibodies. Subtypes H, L and T are expressed at different temperatures [6], [8], with subtype H being expressed at “high” temperature (20–35°C), subtype L at “low” temperature (<20°C) [6] and subtype T (torrid) at temperatures above 36°C [8], [9]. Gradual switching of i-ag subtypes occurs when temperatures are shifted [6], [8]. The gene coding for i-ag was named *Ser* after the word “serotype” [10]. So far, up to six subtype H allelic variants, one subtype J gene and six subtype L paralogs were found [11]–[15]. One common characteristic among these Ser proteins is a repetitive cysteine-rich motif [13], [14]. Such features are also common in surface proteins of other unicellular eukaryotes [16]. The control mechanism of *Ser* expression is not well understood, but the mRNA half-life of *Ser*H3 (normally expressed at 20–35°C) is rapidly decreased when temperature is shifted up to 40°C [17], [18]. Treatment with protein synthesis and protein kinase inhibitors can prolong *Ser*H3 mRNA half-life during this temperature shift, suggesting that there are proteins and phospho-proteins involved in *Ser* mRNA degradation [19]. However, the role of *T. thermophila* i-ag remains unclear, though it may involve sensing the environment or prey-predator recognition, similar to *Paramecium* surface antigen [20].

Even though sequence variation is a hallmark of these highly diverged surface proteins, they often contain repetitive cysteine-rich motifs. The periodic cysteine residues could form disulfide bonds in a consistent pattern among proteins in the same family, but there is no experimental data on the formation of disulfide bonds in i-ag at this point. The existence of the disulfide bonds might introduce an extremely hydrophobic moiety at the core of protein [21]. It was suggested that 'hydrophobic collapse' might play a crucial role in protein folding in general via hydrophobic core nucleus which drives the folding process [22]. Disulfide bond formation could allow protein to become highly divergent on the surface while maintaining the overall fold. Different number of cysteine pair per one repeating sequence block in different i-ag subtypes has been documented [12]. Despite high sequence variation among *Ser* genes, the pattern of cysteine rich motif is a common feature among them.

Another distinct feature among *Ser* genes is the consensus sequence at the C-terminus specific for glycosylphosphatidylinositol (GPI) anchor modification. *T. thermophila* i-ag subtype H was shown to be GPI-anchored protein by radiolabelling [23], [24]. Putative GPI anchor site was predicted to be located at the C-terminus of the Ser proteins [13], [14]. There is a GPI anchor signal sequence at the C-terminus, which can be recognized by transamidase. GPI anchor signal can be divided into three regions: GPI attachment site (ω site), spacer region of polar residues (ω+3 to ω+8), and hydrophobic region (ω+9 to the C-terminus) [25]. There may be the minimum length for hydrophobic region required for GPI attachment [26]. Because GPI signal has no detectable conserved sequence, it cannot be identified by sequence similarity approach. However, the signal sequences surrounding the GPI attachment site can be defined as regions of amino acid residues with different physical properties such as size and hydrophobicity [25]. This allows the prediction of GPI-anchored protein using knowledge-based algorithm [27].

**Figure 1:**
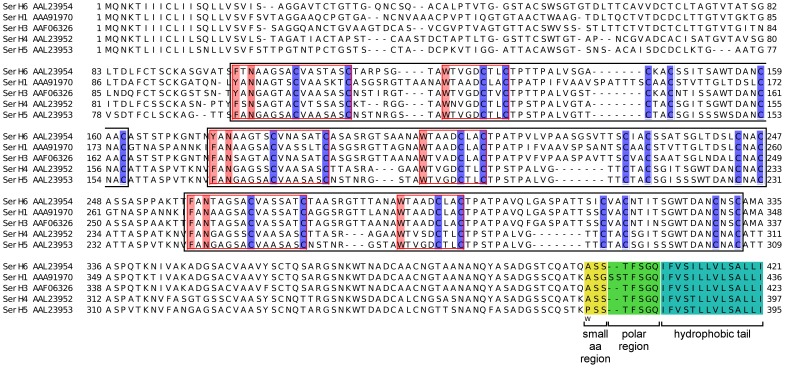
Features of *T. thermophila* Ser protein sequences. Selected known Ser protein sequences are aligned to show their Cys pair, repetitive block and GPI anchor site. Cys residues are highlighted (dark blue). Each repetitive block is indicated by black box. Red box indicates sequence feature which appears in each repetitive block. The region predicted as GPI anchor signal by FragAnchor is color-shaded. Predicted GPI attachment site is marked by letter “w”. According to data from known Ser, number of Cys residues per repetitive block is unique for each subtype (SerL: 6 Cys per block; SerH: 8 Cys per block). The length of each repetitive block differs among various subtypes and is also varied between 55–100 aa. GPI anchor signal predicted by FragAnchor exhibits region of small amino acids (Ala, Gly, Ser) where GPI is attached (yellow), followed by polar region (green) and hydrophobic tail (light blue).

There are 24,725 predicted protein-coding genes in *T. thermophila* MAC genome [28], [29]. The majority of the genes in *T. thermophila* are transcriptionally regulated as determined by nuclear run-on assay [30]. Post-transcriptional control via mRNA stability was also observed in *Ser*H3 gene regulation [18]. As the *Ser* gene family is highly polymorphic, sequence homology analysis alone cannot recognize the full set of putative *Ser* genes. In order to gain a full understanding of the antigenic variation in *T. thermophila Ser* genes, it is necessary to overcome this problem. In this study, the repertoire of the *T. thermophila Ser* gene family was annotated by setting search criteria based on the repetitive cysteine-rich motif and the signal sequence for GPI anchor. Two hundred and sixteen putative *Ser* genes from *T. thermophila* MAC genome sequence were selected including the known *Ser* genes. Previous studies have shown that each *Ser* subtype has a specific number of Cys residues per repetitive block: *Ser*H with 8 Cys residues per block [14], [15], *Ser*J with10 Cys residues per block [12] and *Ser*L with 6 Cys residues per block. The periodic cysteine block pattern, CX_(≥6)_CX_(≥1)_CX_(≥1)_CX_(≥1)_CX_(≥1)_C, and the GPI anchor site were used as the search criteria for the *Ser* gene family (Figure 1 and Figure S1 in File S1) [13]–[15]. Putative *Ser* genes were further characterized based on their phylogenetic distribution with experimentally classified *Ser* subtypes. By combining the data from the *Ser* candidates and the known *Ser* genes, the patterns of *Ser* chromosomal localization and gene expression were revealed.

## Materials and Methods

### Sequence and genome data

The whole predicted protein sequences of *Tetrahymena thermophila* were downloaded from *Tetrahymena* Genome Database (http://ciliate.org/index.php/home/downloads). The 5'-*UTR* sequences of *Ser* candidates were obtained from TetraFGD Genome Browser (http://tfgdgb.ihb.ac.cn). Thirteen known *Ser* subtypes (listed in Table S1 in File S1) from NCBI database were used in this study as reference [28], [29].

### Identification of *Ser* gene candidates

**Figure 2:**
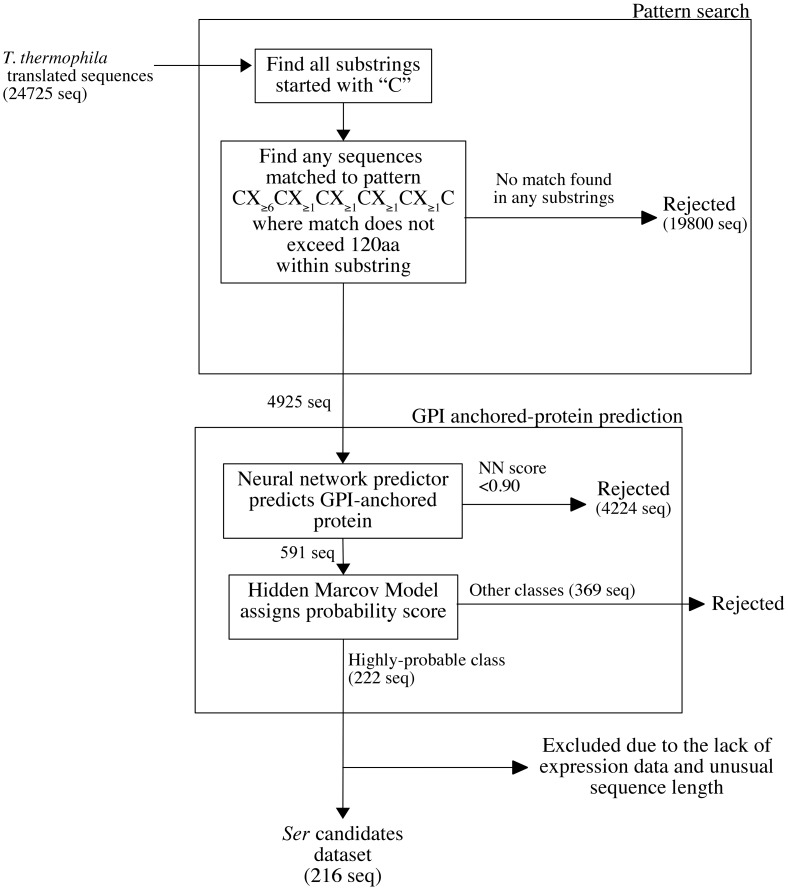
*T. thermophila Ser* prediction algorithm diagram. *T. thermophila* translated sequences were the input data for the pattern search script. *Ser* candidate detection pattern is based on length and number of Cys in a single repetitive block from known *Ser* data. In order to find all possible pattern matches within one protein sequence, the script breaks the input sequence into smaller substrings starting with C (Cys). Then the script finds the match within that substring. *T. thermophila* translated sequences which pass pattern search algorithm were then input into web-based GPI-anchored protein predictor FragAnchor. FragAnchor assigned predicted GPI anchored proteins into classes based on their HMM scores. Sequences assigned into the “highly probable” class were selected as *Ser* candidates.

*Ser* prediction algorithm was composed of two main parts, cysteine pattern search and GPI-anchored protein prediction (Figure 2). Custom Perl script was used to perform the search for the cysteine-rich pattern. The script was set to select any sequence containing CX(_≥_6)CX_(≥1)_CX_(≥1)_CX_(≥1)_CX_(≥1)_C or CX_long_CX_short_C (C  =  cysteine, X  =  any amino acid except cysteine, X_short_  = 1 to 5 residues and X_long_ >5 residues) within 120 amino acid residues. This length of amino acid residues was chosen because it gave the best pattern match to the known *Ser* genes. The results were then refined by GPI-anchored protein prediction employing the web-based program FragAnchor [31]. Hidden Markov Model (HMM) implemented in FragAnchor was used to assign the probability score. Amino acid sequences matching the defined cysteine pattern with highly probable GPI-anchored protein determined by FragAnchor were selected as *Ser* candidates.

### Phylogenetic analysis

The translated sequences of *Ser* candidates were aligned using ClustalX 2.0.12 with default multiple alignment parameters (gap opening penalty  = 10; gap extension penalty  = 0.2; Gonnet series weight matrix). Alignment was then adjusted manually. Neighbor-joining (NJ) tree was calculated with 1000 bootstrap replicates using ClustalX. Protein evolutionary model was selected by ProtTest [32]. Maximum likelihood (ML) tree was estimated using RAxML 7.2.8 as implemented on the CIPRES Science Gateway [33]–[35]. Phylogenetic trees were then created using Dendroscope [36]. TTHERM_01098980 was excluded from phylogenetic analysis because its sequence has an unusual sequence length of 3751 amino acid residues, preventing it from being aligned.

### *Ser* gene expression analysis

*T. thermophila* genome-wide gene expression (growth, starvation and conjugation) microarray data was retrieved from *Tetrahymena* Functional Genomics Database (TetraFGD) (http://tfgd.ihb.ac.cn/) [37]. MultiExperimentViewer (MeV) was employed to evaluate gene expression clusters and to classify the expression pattern into subgroups (www.tm4.org/mev/). Clustering was identified with K-Means clustering module (KMC), and distance was calculated by Pearson correlation.

## Results

### Identification and classification of *Ser* genes

Due to the high degree of polymorphism, identification of *T. thermophila Ser* genes by sequence homology alone is limited by low sequence conservation. In order to systematically search for *Ser* candidates, two criteria were applied based on the common features found in existing i-ag proteins (six *Ser*H, one *Ser*J and six *Ser*L) namely, the presence of Cys residue pattern block CXlongCXshortC and the GPI-anchor signal located at the C-terminus. For the first criterion, a Perl script was set up to search for the *T. thermophila* proteins which, for any window frame containing 4 or 6 Cys residues within 30–120 amino acid residues, the number of amino acid between the first Cys pair is more than 5 and the number of amino acid between the other Cys pairs is at least 1 (Figure 2). After determining the number of hits versus search pattern for saturation in gene numbers and change in phylogenetic pattern, the search criterion was limited to the minimum of 6 Cys residues within a 120 amino acid interval. Any protein containing CX(_≥6_)CX_(≥1)_CX_(≥1)_CX_(≥1)_CX_(≥1)_C sequence within 120 amino acid residues was selected, resulting in 4,925 hits out of 24,725 *T. thermophila* predicted proteins. For the second selection criterion, FragAnchor classified 216 Ser candidates as “highly probable” GPI-anchored proteins including all known *Ser* genes (Figure 1–2 and Figure S1 in File S1). Sequences classified as “probable” or “weakly-probable” GPI-anchored proteins were excluded in order to avoid false positives. The approach successfully identified all four annotated *Ser* genes in the genome of *T. thermophila* strain SB210 with perfect identity match to the experimentally identified *Ser* genes.

### Classification of putative *Ser* genes from *T. thermophila* strain SB210

**Figure 3:**
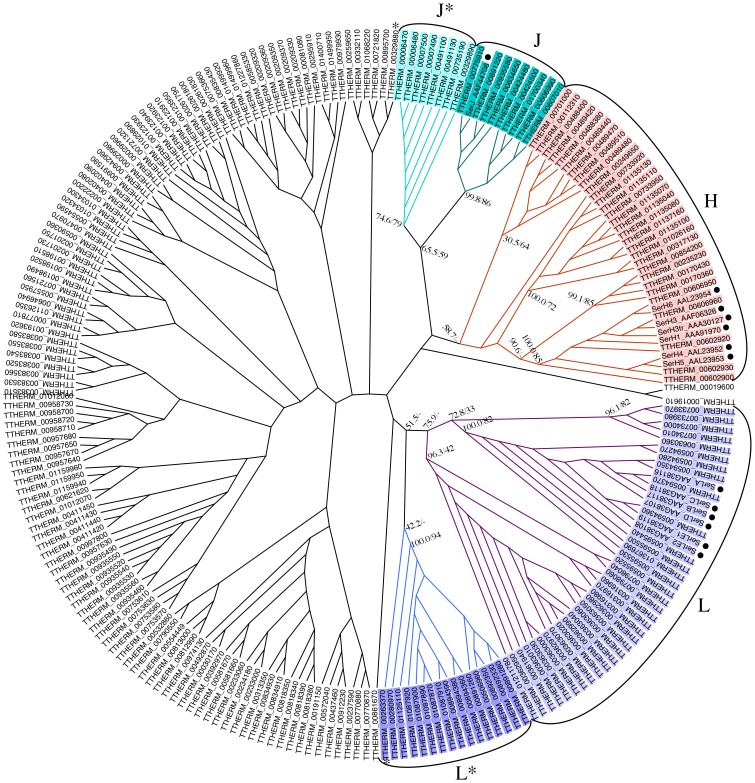
Neighbor-joining cladogram of *Ser* candidates identified in this study. Known Ser proteins are also included in this figure (black dots). 100-replicate bootstrapping was performed. Group-assigned candidates are highlighted. Bootstrap support percentages for the Neighbor-Joining (NJ) tree and the Maximum Likelihood (ML) tree (dash indicates undetermined bootstrap support value) are respectively shown on selected branch nodes. Among group-assigned candidates, TTHERM_00263370 and TTHERM_00329880 (marked with asterisk) are inconsistent between NJ and ML trees. In NJ tree, TTHERM_00263370 is clustered with L* clade. But in ML tree, it is closer to *Ser*L clade. TTHERM_00329880 is clustered with *Ser*H clade in the ML tree but not in the NJ tree.

Forty-five percent of the selected genes could be grouped with three identified *Ser* subtypes, *Ser*H, *Ser*L and *Ser*J, with good bootstrap support from NJ analyses (*Ser*H: 58.7% NJ bootstrap support; *Ser*L: 72.8% NJ bootstrap support; and *Ser*J: 99.8% NJ bootstrap support). Two distinct branches of *Ser* candidates, grouped with *Ser*J and *Ser*L, were found and were named J* (74.6% NJ bootstrap support) and L* (42.2% NJ bootstrap support) to reflect their phylogenetic association with *Ser*J and *Ser*L groups, respectively (Figure 3). Candidates grouped with the known *Ser* subtypes are listed in Table S2 in File S1.

### *Ser* chromosomal location

**Figure 4:**
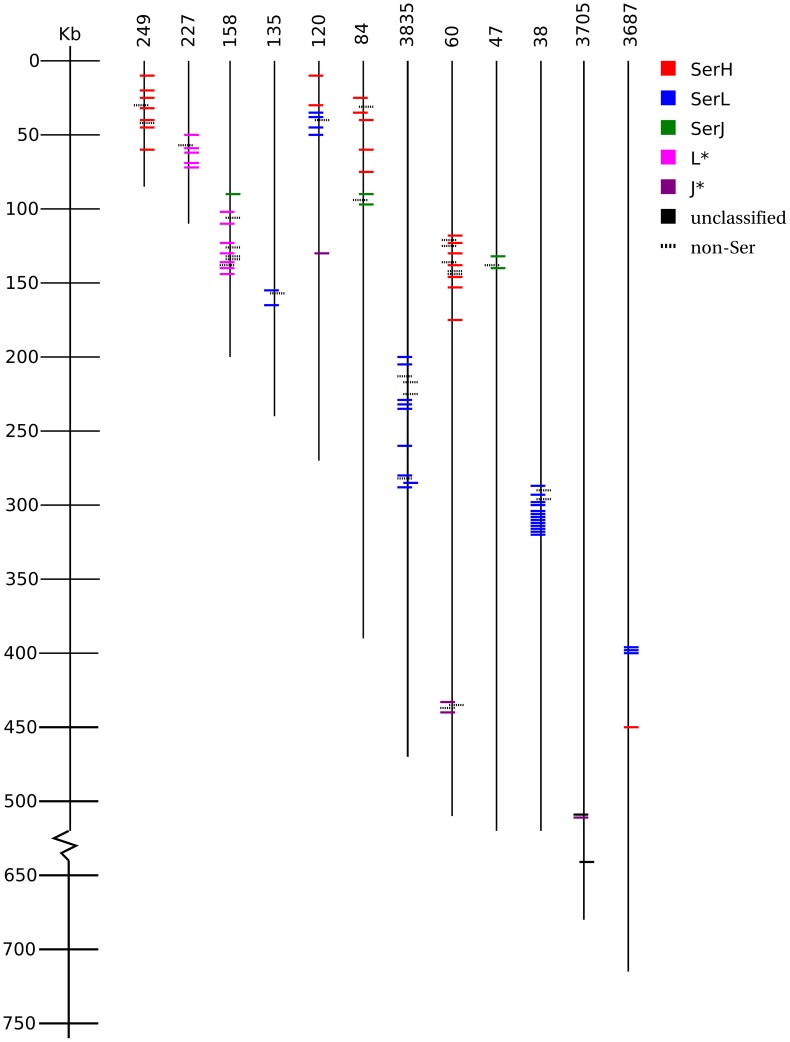
Distribution of *Ser* candidates on *T. thermophila* MAC scaffold. Each short horizontal line represents one *Ser* gene. *Ser* subtype is color-coded. Dash line represents non-*Ser* gene which locates within *Ser* tandem arrays. Gene orientation on plus or minus strand is depicted by left or right alignment respectively. Scaffold number is shown on the top of each scaffold line (vertical line). Only scaffolds containing classified *Ser* tandem array are shown.

Chromosomal location analysis focused on the newly identified *Ser* genes with known subtypes. They were dispersed on 76 out of the estimated 250–300 MAC chromosomes in *T. thermophila*. Figure 4 shows the distribution of subtype-classified *Ser* candidates on 12 scaffolds, with the majority (86%) of the identified *Ser* candidate genes located in close proximity to one another. *Ser* genes tend to form a tandem array of the same subtype (Table S2 in File S1). Six MAC scaffolds contained tandem arrays with only one subtype of *Ser* genes, and those in close proximity on the same scaffold tended to have the same orientation, with a few exceptions, such as subtype-H TTHERM_00602920 on scaffold 84 and subtype-L TTHERM_00595520 on scaffold 3835. There appears to be no preference for scaffold size or chromosomal region where *Ser* genes are located.

### *Ser* gene expression analysis

**Figure 5:**
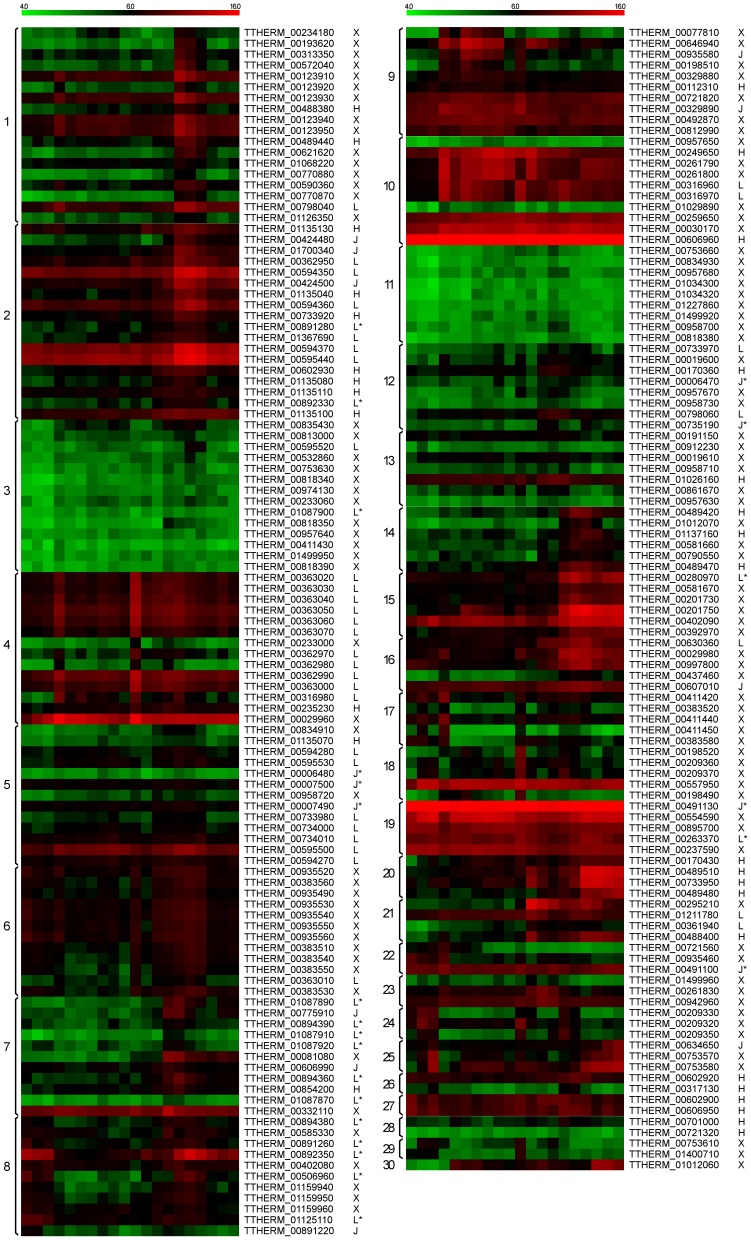
*Ser* gene expression clusters. Each row represents one gene. Gene ID and subtype were listed. Unclassified *Ser* candidates are marked as X. Gene expression data was subjected to K-means clustering method using Pearson correlation to measure distance. Expression cluster ID is indicated as arabic number on the left of expression heatmap. Scale bar represents log2-transformed gene expression value. Red indicates expression value above median, and green indicates expression value below median. Clustering analysis was performed using MeV 4.7.

**Figure 6:**
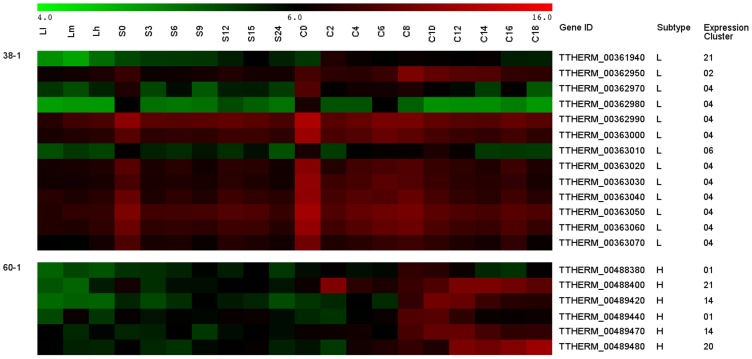
Expression profile of *Ser* genes from tandem array 38-1 and 60-1. Gene expression data was collected from *T. thermophila* during growth at low (Ll), medium (Lm), and high (Lh) densities. The data from starvation (S0, S3, S6, S9, S12, S15, S24) and conjugation (C0, C2, C4, C6, C8, C10, C12, C14, C16, C18) samples was also included with numerical values showing hours in each particular condition [37]. Complete *Ser* expression cluster result is available in Table S2 and S3 in File S1.

*Ser* expression patterns during growth, starvation and conjugation were analyzed based on DNA microarray data [37]. *Ser* expression data could be grouped into 30 clusters based on their expression patterns (Figure 5). No correlation was found between specific *Ser* subtypes and expression patterns. Certain *Ser* tandem genes were associated with the same expression pattern. For example, ten out of thirteen subtype-L *Ser* genes on scaffold 38 were found in expression cluster 4 (Figure 6, upper panel). However, some tandem arrays were not in the same expression cluster, but their expression patterns appeared to be stage-specific. For example, scaffold 60 contains a tandem array of 6 subtype-H genes grouped into four different expression clusters (Figure 6, lower panel). Interestingly, they were all up-regulated during conjugation, but at different time points. Data of similar analyses on locations and expression patterns of unclassified *Ser* candidates are available in Table S3 in File S1.

## Discussion

*T. thermophila* i-ag was originally identified as variant surface antigen. Their high degree of polymorphism has prevented systematic identification of its *Ser* family based on sequence homology alone despite available genomic data. In this study, an alternative strategy was adopted for identifying this gene family by selecting two common features of the known *Ser* genes, namely, the Cys-rich motif and GPI-anchor site. Their phylogenetic distributions were analyzed in order to characterize *Ser* candidates that are related to the known subtypes. The approach identified *Ser* candidates that can be grouped into the known subtypes. However, 55% of the genes could not be classified into any known subtype based on phylogenetic analysis, and might belong to new subtypes. At present, the genes encoding several types of surface antigens such as *SerT*, *SerS*, *SerM* and *SerI* have not been identified indicating the existence of more *Ser* repertoires and subtypes [38]. In addition, certain unclassified gene candidates exhibit properties similar to *Ser*, such as tendency to be located in tandem and have similar gene expression profile. It is possible that the missing *Ser* subtypes might belong to one of the unclassified gene families. Experiments with specific antibodies to Ser candidates will be required in order to prove that they are indeed i-ag proteins. Sequence comparison and synteny analysis of the highly polymorphic *Ser* genes from more *T. thermophila* isolates will confirm whether these genes are under positive selection which is a strong evolutionary driving force for mating proteins, molecular sensors and evasive decoys [39], [40]. Using the Ser candidates as blastp query on NCBI protein database yields sixteen *T. thermophila* proteins not previously included in the list [Table S4 in File S1]. Nevertheless, they either lack the GPI anchor motif or matching cysteine pattern. The phylogenetic analysis showed that these proteins are not grouped with any *Ser* candidate. The analysis outside *T. thermophila* revealed two weak hits with the proteins from other ciliates [*Ichthyophthirius multifiliis* AAK94941 with 21% identity to *SerL* and *Paramecium tetraurelia* XP_001450224 with 22% identity to *SerH*]. This might indicate that the *Ser* gene family is unique to *T. thermophila*.

i-ag proteins were discovered based on their variation in response to antibodies directed at *T. thermophila* surface antigens. This is a hallmark for many antigenic variation phenomena among parasitic and free-living protozoa [41]. In parasitic protozoa such as *Plasmodium falciparum*, a family of proteins on infected red blood cells is needed for a parasitic adherence mechanism to human cells and tissue which is crucial for malaria pathogenesis [42]. Antigenic variation in *P. falciparum* switches the variant pathogenic proteins in order to avoid immune detection [43]. Other parasitic protozoa also exploit a similar system [44], [45]. When antigenic variation is compromised, the parasite becomes vulnerable to the immune system [46].

Our study took advantage of the available *T. thermophila* MAC genome and DNA microarray expression data to analyze the *Ser* gene family. These *Ser* genes are organized in tandem arrays on several MAC scaffolds. These tandem arrays often belong to the same subtype, suggesting that they arose by gene duplication or genetic recombination. Expression pattern cluster analysis does not explicitly indicate the role of the *Ser* gene family in any developmental stage in particular. Available microarray data also did not include every known culture conditions for inducing the expression of *Ser* genes. However, stage-specific expression patterns of several *Ser* transcripts at the same time point were observed, and thus mutual exclusion mechanism is not likely the only strategy underlying expression control of every *Ser* gene.

Unlike parasitic protozoa, free-living protozoa are not subject to host immune pressure, and the purpose for having surface antigenic variation remains unclear. Nevertheless, understanding the mechanism of antigenic variation in free-living organisms could provide new insights into the evolution and regulation control of antigenic variation in parasitic organisms. Thus identifying the whole repertoire of the *Ser* gene family is the first step towards the exploration of antigenic variation in *T. thermophila*, an important model organism for many seminal discoveries in molecular biology.

## Supporting Information

File S1 **Supporting information.**
(DOC)
