# Supplementary material for: Genome-Wide Prediction of the Polymorphic Ser Gene Family in Tetrahymena thermophila Based on Motif Analysis
Source: PLoS One. 2014 Aug 18;9(8):e105201. doi: 10.1371/journal.pone.0105201 (PMC4136848; doi:10.1371/journal.pone.0105201)

**Table S1** Previously identified *Ser* genes. Known *Ser* genes were used in this study as the references for subtype classification. Four of the *Ser* candidates picked up by our search criteria are identical matches to known *Ser* genes (marked by *).

| Accession | Uniprot Entry | Subtype |
| --- | --- | --- |
| AAA91970 | Q27197 | SerH |
| AAF06326* | Q9U697 | SerH |
| AAA30127 | Q94827 | SerH |
| AAL23952 | Q95V71 | SerH |
| AAL23953 | Q95V70 | SerH |
| AAL23954 | Q95V69 | SerH |
| AAF44706 | Q9NGR2 | SerJ |
| AAG38116* | Q9GPP4 | SerL |
| AAG38117 | Q9GPP3 | SerL |
| AAG38118* | Q9GPP2 | SerL |
| AAG38107 | Q9GPP0 | SerL |
| AAG38119* | Q9GPP1 | SerL |
| AAG38108 | Q9GPN9 | SerL |

**Table S2** Gene ID of subtype-classified *Ser* candidates listed by their location and expression cluster. Unclassified *Ser* candidates located on the same MAC scaffold are also shown and marked as X. Location is indicated in ####-# format with the first four digits representing the scaffold ID and the last digit indicating the numerical code of the tandem array. Tandem array is defined as an array of *Ser* genes located no further than 10 kb from other *Ser* genes. Expression cluster as determined by K-means clustering method (KMC) for each *Ser* candidate is also listed.

| Gene ID | Location | Subtype | Expression Cluster |
| --- | --- | --- | --- |
| TTHERM_00006470 | 1-1 | J* | 12 |
| TTHERM_00006480 | 1-1 | J* | 05 |
| TTHERM_00007490 | 1-1 | J* | 05 |
| TTHERM_00007500 | 1-1 | J* | 05 |
| TTHERM_00019600 | 1-2 | X | 12 |
| TTHERM_00019610 | 1-2 | X | 13 |
| TTHERM_00733920 | 120-1 | SerH | 02 |
| TTHERM_00733950 | 120-2 | SerH | 20 |
| TTHERM_00733970 | 120-2 | SerL | 12 |
| TTHERM_00733980 | 120-2 | SerL | 05 |
| TTHERM_00734000 | 120-2 | SerL | 05 |
| TTHERM_00734010 | 120-2 | SerL | 05 |
| TTHERM_00735190 | 120-3 | J* | 12 |
| TTHERM_00798040 | 135-1 | SerL | 01 |
| TTHERM_00798060 | 135-1 | SerL | 12 |
| TTHERM_00170360 | 14-1 | SerH | 12 |
| TTHERM_00170430 | 14-2 | SerH | 20 |
| TTHERM_00891220 | 158-1 | SerJ | 08 |
| TTHERM_00891260 | 158-2 | L* | 08 |
| TTHERM_00891280 | 158-2 | L* | 02 |
| TTHERM_00892330 | 158-3 | L* | 02 |
| TTHERM_00892350 | 158-3 | L* | 08 |
| TTHERM_00894360 | 158-3 | L* | 07 |
| TTHERM_00894380 | 158-3 | L* | 08 |
| TTHERM_00894390 | 158-3 | L* | 07 |
| TTHERM_00935460 | 169-1 | X | 22 |
| TTHERM_00935490 | 169-1 | X | 06 |
| TTHERM_00935520 | 169-1 | X | 06 |
| TTHERM_00935530 | 169-1 | X | 06 |
| TTHERM_00935540 | 169-1 | X | 06 |
| TTHERM_00935550 | 169-1 | X | 06 |
| TTHERM_00935560 | 169-1 | X | 06 |
| TTHERM_00935580 | 169-2 | SerJ | 09 |
| TTHERM_00233000 | 19-1 | X | 04 |
| TTHERM_00233060 | 19-2 | X | 03 |
| TTHERM_00234180 | 19-3 | X | 01 |
| TTHERM_00235230 | 19-4 | SerH | 04 |
| TTHERM_01087870 | 227-1 | L* | 07 |
| TTHERM_01087890 | 227-1 | L* | 07 |
| TTHERM_01087900 | 227-1 | L* | 03 |
| TTHERM_01087910 | 227-1 | L* | 07 |
| TTHERM_01087920 | 227-1 | L* | 07 |
| TTHERM_00259650 | 23-1 | X | 10 |
| TTHERM_00261790 | 23-2 | X | 10 |
| TTHERM_00261800 | 23-2 | X | 10 |
| TTHERM_00261830 | 23-2 | X | 23 |
| TTHERM_00263370 | 23-3 | L* | 19 |
| TTHERM_01135040 | 249-1 | SerH | 02 |
| TTHERM_01135070 | 249-2 | SerH | 05 |
| TTHERM_01135080 | 249-2 | SerH | 02 |
| TTHERM_01135100 | 249-2 | SerH | 02 |
| TTHERM_01135110 | 249-2 | SerH | 02 |
| TTHERM_01135130 | 249-2 | SerH | 02 |
| TTHERM_01137160 | 249-3 | SerH | 14 |
| TTHERM_00316960 | 3687-1 | SerL | 10 |
| TTHERM_00316970 | 3687-1 | SerL | 10 |
| TTHERM_00316980 | 3687-1 | SerL | 04 |
| TTHERM_00317130 | 3687-2 | SerH | 26 |
| TTHERM_00329880 | 3705-1 | X | 09 |
| TTHERM_00329890 | 3705-1 | SerJ | 09 |
| TTHERM_00332110 | 3705-2 | X | 07 |
| TTHERM_00361940 | 38-1 | SerL | 21 |
| TTHERM_00362950 | 38-1 | SerL | 02 |
| TTHERM_00362970 | 38-1 | SerL | 04 |
| TTHERM_00362980 | 38-1 | SerL | 04 |
| TTHERM_00362990 | 38-1 | SerL | 04 |
| TTHERM_00363000 | 38-1 | SerL | 04 |
| TTHERM_00363010 | 38-1 | SerL | 06 |
| TTHERM_00363020 | 38-1 | SerL | 04 |
| TTHERM_00363030 | 38-1 | SerL | 04 |
| TTHERM_00363040 | 38-1 | SerL | 04 |
| TTHERM_00363050 | 38-1 | SerL | 04 |
| TTHERM_00363060 | 38-1 | SerL | 04 |
| TTHERM_00363070 | 38-1 | SerL | 04 |
| TTHERM_00112310 | 3812-1 | SerH | 09 |
| TTHERM_00123910 | 3812-2 | X | 01 |
| TTHERM_00123920 | 3812-2 | X | 01 |
| TTHERM_00123930 | 3812-2 | X | 01 |
| TTHERM_00123940 | 3812-2 | X | 01 |
| TTHERM_00123950 | 3812-2 | X | 01 |
| TTHERM_00594270 | 3835-1 | SerL | 05 |
| TTHERM_00594280 | 3835-1 | SerL | 05 |
| TTHERM_00594350 | 3835-1 | SerLA | 02 |
| TTHERM_00594360 | 3835-2 | SerLE1 | 02 |
| TTHERM_00594370 | 3835-2 | SerLC | 02 |
| TTHERM_00595440 | 3835-3 | SerL | 02 |
| TTHERM_00595500 | 3835-4 | SerL | 05 |
| TTHERM_00595520 | 3835-4 | SerL | 03 |
| TTHERM_0059530 | 3835-4 | SerL | 05 |
| TTHERM_00424480 | 47-1 | SerJ | 02 |
| TTHERM_00424500 | 47-1 | SerJ | 02 |
| TTHERM_00488380 | 60-1 | SerH | 01 |
| TTHERM_00488400 | 60-1 | SerH | 21 |
| TTHERM_00489420 | 60-1 | SerH | 14 |
| TTHERM_00489440 | 60-1 | SerH | 01 |
| TTHERM_00489470 | 60-1 | SerH | 14 |
| TTHERM_00489480 | 60-1 | SerH | 20 |
| TTHERM_00489510 | 60-2 | SerH | 20 |
| TTHERM_00491100 | 60-3 | J* | 22 |
| TTHERM_00491130 | 60-3 | J* | 19 |
| TTHERM_00602900 | 84-1 | SerH | 27 |
| TTHERM_00602920 | 84-1 | SerH | 26 |
| TTHERM_00602930 | 84-1 | SerH | 02 |
| TTHERM_00606950 | 84-2 | SerH | 27 |
| TTHERM_00606960 | 84-3 | SerH | 10 |
| TTHERM_00606990 | 84-4 | SerJ | 07 |
| TTHERM_00607010 | 84-4 | SerJ | 16 |
| TTHERM_00701000 | 109 | SerH | 28 |
| TTHERM_00775910 | 129 | SerJ | 07 |
| TTHERM_00854200 | 151 | SerH | 07 |
| TTHERM_00861670 | 153 | L* | 13 |
| TTHERM_01026160 | 202 | SerH | 13 |
| TTHERM_00249650 | 22 | SerH | 10 |
| TTHERM_01125110 | 245 | L* | 08 |
| TTHERM_01211780 | 275 | SerL | 21 |
| TTHERM_01367690 | 350 | SerL | 02 |
| TTHERM_00280970 | 3694 | L* | 15 |
| TTHERM_00506960 | 3723 | L* | 08 |
| TTHERM_00630360 | 3831 | SerL | 16 |
| TTHERM_01700340 | 573 | SerJ | 02 |
| TTHERM_00634650 | 90 | SerJ | 25 |

**Table S3** Gene ID of unclassified *Ser* candidates listed by their location and expression cluster. The table includes the rest of the unclassified *Ser* candidates with the same labeling system in in Supplementary Table 2.

| Gene ID | Location | Subtype | Expression Cluster |
| --- | --- | --- | --- |
| TTHERM_00721320 | 115-1 | X | 28 |
| TTHERM_00721560 | 115-1 | X | 22 |
| TTHERM_00721820 | 115-2 | X | 09 |
| TTHERM_00753570 | 122-1 | X | 25 |
| TTHERM_00753580 | 122-1 | X | 25 |
| TTHERM_00753610 | 122-2 | X | 29 |
| TTHERM_00753630 | 122-2 | X | 03 |
| TTHERM_00753660 | 122-2 | X | 11 |
| TTHERM_00770870 | 127-1 | X | 01 |
| TTHERM_00770880 | 127-1 | X | 01 |
| TTHERM_00812990 | 138-1 | X | 09 |
| TTHERM_00813000 | 138-1 | X | 03 |
| TTHERM_00818340 | 139-1 | X | 03 |
| TTHERM_00818350 | 139-1 | X | 03 |
| TTHERM_00818380 | 139-1 | X | 11 |
| TTHERM_00818390 | 139-1 | X | 03 |
| TTHERM_00834910 | 145-1 | X | 05 |
| TTHERM_00834930 | 145-1 | X | 11 |
| TTHERM_00835430 | 145-2 | X | 03 |
| TTHERM_00198490 | 17-1 | X | 18 |
| TTHERM_00198510 | 17-1 | X | 09 |
| TTHERM_00198520 | 17-1 | X | 18 |
| TTHERM_00201730 | 17-2 | X | 15 |
| TTHERM_00201750 | 17-3 | X | 15 |
| TTHERM_00209320 | 17-4 | X | 24 |
| TTHERM_00209330 | 17-4 | X | 24 |
| TTHERM_00209350 | 17-4 | X | 24 |
| TTHERM_00209360 | 17-4 | X | 18 |
| TTHERM_00209370 | 17-4 | X | 18 |
| TTHERM_00957630 | 177-1 | X | 13 |
| TTHERM_00957640 | 177-1 | X | 03 |
| TTHERM_00957650 | 177-1 | X | 10 |
| TTHERM_00957670 | 177-2 | X | 12 |
| TTHERM_00957680 | 177-2 | X | 11 |
| TTHERM_00958700 | 177-3 | X | 11 |
| TTHERM_00958710 | 177-3 | X | 13 |
| TTHERM_00958720 | 177-3 | X | 05 |
| TTHERM_00958730 | 177-3 | X | 12 |
| TTHERM_01012060 | 196-1 | X | 30 |
| TTHERM_01012070 | 196-1 | X | 14 |
| TTHERM_01034300 | 205-1 | X | 11 |
| TTHERM_01034320 | 205-1 | X | 11 |
| TTHERM_00402080 | 3713-1 | X | 08 |
| TTHERM_00402090 | 3713-1 | X | 15 |
| TTHERM_00411420 | 3714-1 | X | 17 |
| TTHERM_00411430 | 3714-1 | X | 03 |
| TTHERM_00411440 | 3714-1 | X | 17 |
| TTHERM_00411450 | 3714-1 | X | 17 |
| TTHERM_00313350 | 3810-1 | X | 01 |
| TTHERM_00572040 | 3810-2 | X | 01 |
| TTHERM_01159940 | 3823-1 | X | 08 |
| TTHERM_01159950 | 3823-1 | X | 08 |
| TTHERM_01159960 | 3823-1 | X | 08 |
| TTHERM_00029960 | 3825-1 | X | 04 |
| TTHERM_00029980 | 3825-1 | X | 16 |
| TTHERM_00030170 | 3825-2 | X | 10 |
| TTHERM_00581660 | 3827-1 | X | 14 |
| TTHERM_00581670 | 3827-1 | X | 15 |
| TTHERM_00077810 | 3828-1 | X | 09 |
| TTHERM_00081080 | 3828-2 | X | 07 |
| TTHERM_00383510 | 40-1 | X | 06 |
| TTHERM_00383520 | 40-1 | X | 17 |
| TTHERM_00383530 | 40-1 | X | 06 |
| TTHERM_00383540 | 40-1 | X | 06 |
| TTHERM_00383550 | 40-1 | X | 06 |
| TTHERM_00383560 | 40-1 | X | 06 |
| TTHERM_00383580 | 40-1 | X | 17 |
| TTHERM_01499920 | 425-1 | X | 11 |
| TTHERM_01499950 | 425-2 | X | 03 |
| TTHERM_01499960 | 425-2 | X | 23 |
| TTHERM_00790550 | 133 | X | 14 |
| TTHERM_00895700 | 159 | X | 19 |
| TTHERM_00193620 | 16 | X | 01 |
| TTHERM_00912230 | 162 | X | 13 |
| TTHERM_00942960 | 172 | X | 23 |
| TTHERM_00974130 | 181 | X | 03 |
| TTHERM_00997800 | 188 | X | 16 |
| TTHERM_01029890 | 204 | X | 10 |
| TTHERM_01068220 | 217 | X | 01 |
| TTHERM_01126350 | 246 | X | 01 |
| TTHERM_01227860 | 282 | X | 11 |
| TTHERM_00585330 | 3675 | X | 08 |
| TTHERM_01400710 | 368 | X | 29 |
| TTHERM_00191150 | 3697 | X | 13 |
| TTHERM_00237590 | 3706 | X | 19 |
| TTHERM_00437460 | 3708 | X | 16 |
| TTHERM_00492870 | 3711 | X | 09 |
| TTHERM_00590360 | 3726 | X | 01 |
| TTHERM_00646940 | 3727 | X | 09 |
| TTHERM_00557950 | 3735 | X | 18 |
| TTHERM_00392970 | 3813 | X | 15 |
| TTHERM_00532860 | 3830 | X | 03 |
| TTHERM_00554590 | 72 | X | 19 |
| TTHERM_00621620 | 86 | X | 01 |
| TTHERM_00295910 | 92 | X | 21 |

**Table S4** Sixteen *T. thermophila* proteins found by blastp against NCBI protein database using *Ser* candidates as query. Each protein was subjected to GPI-anchored protein prediction program (FragAnchor) and was classified accordingly to probability score using HMM ('Highly probable', 'Probable', 'Weakly probable', ‘HMM score < 0.20’). Protein without GPI signal recognized by FragAnchor was rejected before probability scoring.

| Accesssion | Gene ID | Predicted GPI anchor classification |
| --- | --- | --- |
| XP_001009023 | TTHERM_00260760 | Highly probable |
| XP_001015544 | TTHERM_00383590 | Highly probable |
| XP_001015023 | TTHERM_00675510 | Highly probable |
| XP_001015019 | TTHERM_00673490 | Highly probable |
| XP_001008873 | TTHERM_00187070 | Highly probable |
| XP_001015026 | TTHERM_00675540 | Probable |
| XP_001015018 | TTHERM_00673480 | Probable |
| XP_001015017 | TTHERM_00673470 | Probable |
| XP_001012443 | TTHERM_01031270 | HMM score < 0.20 |
| XP_001029448 | TTHERM_01503030 | HMM score < 0.20 |
| XP_001024289 | TTHERM_00994410 | Rejected |
| XP_001028396 | TTHERM_02444130 | Rejected |
| XP_001007125 | TTHERM_00209340 | Rejected |
| XP_001016147 | TTHERM_00818400 | Rejected |
| XP_001012445 | TTHERM_01032280 | Rejected |
| XP_001033063 | TTHERM_00471790 | Rejected |

**Figure S1** *T. thermophila* Ser protein sequences from subtype L (A) and J (B). The label and color are consistent with Figure 1. The cysteine motif pattern and GPI anchor sites are consistent with the subtype H family in Figure 1.


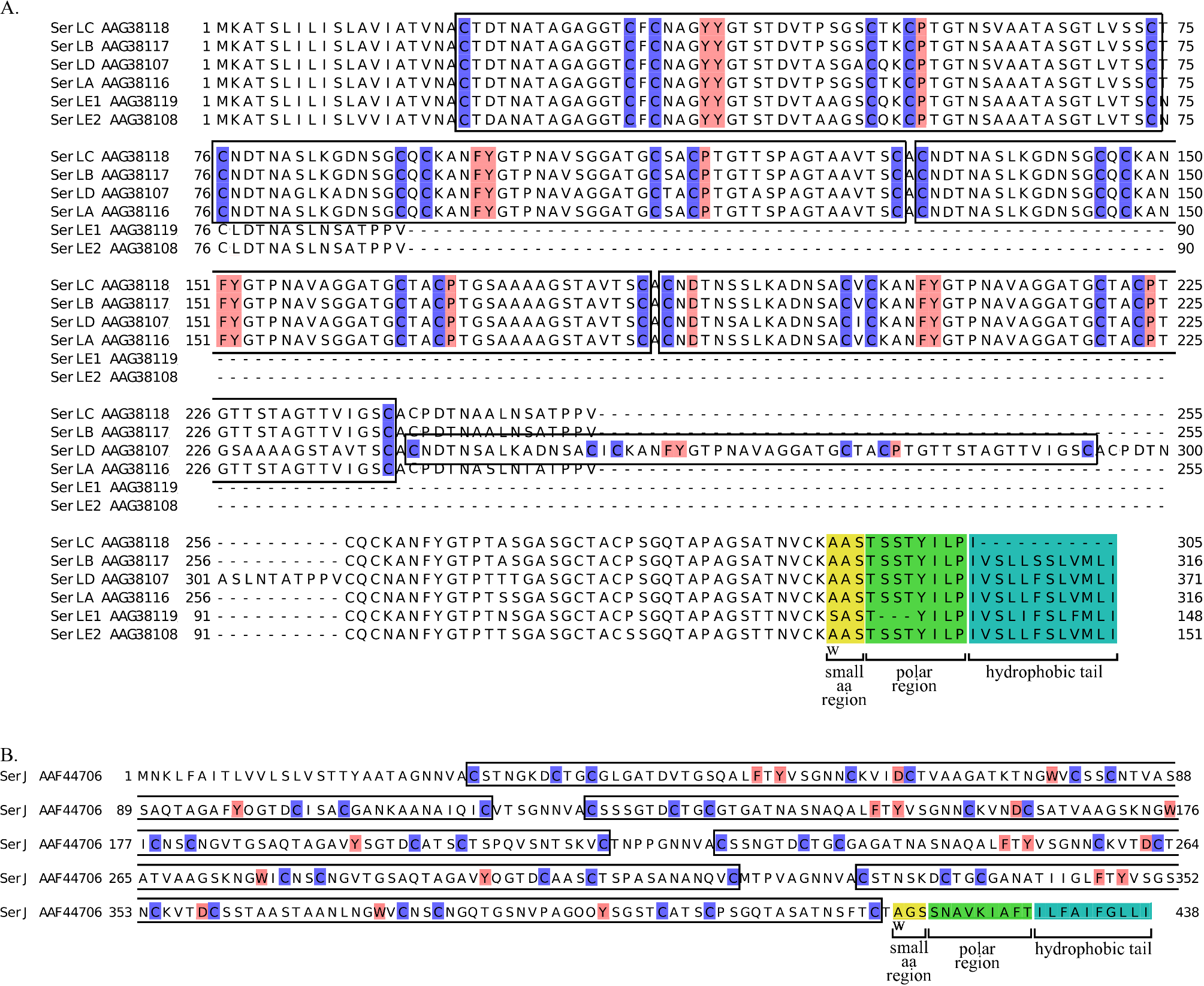

Supplement: File S1 — Supporting information. (DOC) [file pone.0105201.s001.doc]
